# Supplementary material for: Extraction of relations between genes and diseases from text and large-scale data analysis: implications for translational research
Source: BMC Bioinformatics. 2015 Feb 21;16:55. doi: 10.1186/s12859-015-0472-9 (PMC4466840; doi:10.1186/s12859-015-0472-9)
Supplement: Additional file 1: Table S1. — Statistics of the EU-ADR and GAD corpora. The Association type classifies the association according to the level of certainty: TRUE (positive (PA), negative (NA) and speculative (SA)) and FALSE (FA). [file 12859_2015_472_MOESM1_ESM.docx]

**Additional file 1: Table S1.**

| **Association type** | | **EU-ADR** | | | **GAD** |
| --- | --- | --- | --- | --- | --- |
|  |  | **Drug-Disease** | **Target-Drug** | **Gene-Disease** |  |
| **TRUE** | PA | 162 | 157 | 213 | 1833 |
|  | NA | 2 | 6 | 19 | 967 |
|  | SA | 12 | 14 | 30 | - |
| **FALSE** | | 68 | 70 | 93 | 2529 |
| **TOTAL** | | 244 | 247 | 355 | 5329 |
