# Supplementary material for: Extraction of relations between genes and diseases from text and large-scale data analysis: implications for translational research
Source: BMC Bioinformatics. 2015 Feb 21;16:55. doi: 10.1186/s12859-015-0472-9 (PMC4466840; doi:10.1186/s12859-015-0472-9)
Supplement: Additional file 2: — In addition, this article contains supplementary information available online ( http://ibi.imim.es/befree/#supplmaterial ). [file 12859_2015_472_MOESM2_ESM.docx]

# Additional file 2

# “Extraction of relations between genes and diseases from text and large-scale data analysis: implications for translational research”

# À. Bravo, J. Piñero, N. Queralt, M. Rautschka, L.I. Furlong.

Evaluation of the RE system on the AIMED corpus 1

AIMed corpus 1

Evaluation of Kernel based Relation Extraction 2

Results 2

Survey of NER systems 3

Table 1: Selected results obtained by 10-fold cross-validation on the AIMed corpus. 5

References 5

##

## Evaluation of the RE system on the AIMED corpus

## AIMed corpus

The AIMed corpus is widely used for PPI extraction (ftp://ftp.cs.utexas.edu/pub/mooney/bio-data/). The AImed corpus consists of 225 MEDLINE abstracts, of which 200 abstracts describe interactions between human proteins and 25 do not refer to any interaction. There are 5625 annotated sentences, 1008 containing a true PPI (TRUE) and 4617 not containing a true PPI (FALSE).

## Evaluation of Kernel based Relation Extraction

The performance of each model for association classification was evaluated by sentence-level 10-fold cross validation in each corpus. The classifiers’ performances were assessed using P, R and F-score over the class TRUE. TRUE sentences contain real relationships between the entities analysed, in contrast with FALSE sentences where the two entities co-occur, but there is no semantic relationship between them.

## Results

The first experiments were conducted on the AIMed corpus in order to evaluate the performance of the *K_DEP_* kernel with different features and compare it with previous results (Table 1). Compared to the performance obtained with the original system (*K_SL_* with sparse bigrams, P 52.9%, R 65.3%, F 58.1 %), *K_DEP_* alone does not show an improvement in performance (26: P 41.3%, R 61.2%, F 49%). However, the combination of *K_SL_* and *K_DEP_* kernels results in an improvement of the results both at the level of precision and recall. For example, using role, lemma and stem as features leads to high levels of F-score (96: P 55.5%, R 67.2%, F 60.3%; 98: P 55.3%, R 67.3%, F 60.3%). Although we show experiments where the features on the token for the v-walk and e-walk are tested one at a time, we also conducted experiments testing different combinations of these features, but the results were not better than the ones obtained with single features over the token (results not shown). Thus, the use of shallow linguistic information but also syntactic information in the form of dependency walk features lead to more accurate models for PPI relation extraction. Our results are comparable to the results obtained with state-of-the-art approaches tested on the AIMED corpus [1].

## Survey of NER systems

During the initial phase of the project, we evaluated several NER tools publicly available. Our requirement was that the NER tool: a) has to be able to detect and normalize to database identifiers two types of entities: genes/proteins and diseases, b) handles the ambiguities between genes and diseases. After an initial evaluation, the decision was to develop our own system because none of the tools evaluated worked properly for our needs. Another reason for developing our own tool was to be able to regularly update the dictionaries used by the NER to keep the data up-to-date, and to perform a curation of the dictionaries to reduce errors. We also invested efforts on the problem of the ambiguities between entities, which we think is not currently addressed by any other tool. We believe that this point is important for subsequent steps in the text-mining workflow, such as the identification of relationships between entities.

From the tools that we initially evaluated, only Metamap was able to detect and normalize gene and disease entities. However, despite the vocabularies used for detection of diseases are very complete and allow detection of diseases from any disease area, the coverage of gene entities by the UMLS Metathesaurus was not optimal for us and we decided to use our own NER based in our own dictionaries developed by integrating different databases and followed by a semi-automatic curation process.

Below we give a short description of some of the additional NER tools tested:

**Penn BioTagger**

[http://www.seas.upenn.edu/~strctlrn/BioTagger/BioTagger.html](http://www.seas.upenn.edu/%7Estrctlrn/BioTagger/BioTagger.html)

a) Identifies three types of entities: gene entities, genomic variations entities and malignancy type entities.

b) Does not perform entity normalization, focused only in one disease class (cancer)

**AIIA-GMT**

<https://metacpan.org/release/AIIA-GMT>

a) The AIIA gene mention tagger is aimed at recognizing gene mentions in biomedical articles

b) It is only focused in gene mentions, does not cover diseases, and does not perform entity normalization.

**BANNER Named Entity Recognition System and DNorm system**

<http://banner.sourceforge.net/> <http://www.ncbi.nlm.nih.gov/CBBresearch/Lu/Demo/DNorm/>

a) NER system based on CRF to detect gene and disease mentions

b) Only disease entities are normalized (DNorm system)

**cTakes**

<http://ctakes.apache.org/>

A) Apache clinical Text Analysis and Knowledge Extraction System (cTAKES) is an open-source natural language processing system for information extraction from electronic medical record clinical free-text. Uses the UMLS Metathesaurus.

b) Performs entity normalization, but does not focus on gene and proteins. It is designed to work on electronic health records.

## Table 1: Selected results obtained by 10-fold cross-validation on the AIMed corpus.

The best results achieved are highlighted in bold. The first column indicates the number of the experiment, the second column shows if *K_SL_* is used with (SB)/without (O) sparse bigrams, or if it is not used (-). The next two columns focus on *K_DEP_* features, that can be represented as v-walk and/or e-walk: token (T), stem (S), lemma (L), POS-tag (P), role (R) or none (-). The last columns show the result obtained in each experiment indicating precision (P), recall (R) and f-measure (F1) in percentage (%).

| Num. | *K_SL_* | *K_DEP_* | | AIMed | | |
| --- | --- | --- | --- | --- | --- | --- |
|  |  | v-walk | e-walk | P | R | F1 |
| 1 | O | - | - | 51.4 | 65.9 | 57.4 |
| 2 | SB | - | - | 52.9 | 65.3 | 58.1 |
| 6 | - | R | - | 35.7 | 63.2 | 45.3 |
| 26 | - | R | L | 41.3 | 61.2 | 49.0 |
| 36 | - | R | T | 41.5 | 60.5 | 48.8 |
| 41 | O | R | - | 52.6 | 69.2 | 59.3 |
| 96 | SB | R | L | **55.5** | **67.2** | **60.3** |
| 98 | SB | S | R | 55.3 | 67.3 | 60.3 |
| 103 | SB | S | T | 57.4 | 62.3 | 59.4 |

## References

1. Chowdhury MFM, Lavelli A: **Combining tree structures, flat features and patterns for biomedical relation extraction**. In *EACL ’12 Proc 13th Conf Eur Chapter Assoc Comput Linguist*. Association for Computational Linguistics; 2012:420–429.
