# Supplementary material for: Extraction of relations between genes and diseases from text and large-scale data analysis: implications for translational research
Source: BMC Bioinformatics. 2015 Feb 21;16:55. doi: 10.1186/s12859-015-0472-9 (PMC4466840; doi:10.1186/s12859-015-0472-9)
Supplement: Additional file 3: Table S2. — Evaluation of BeFree and SemRep for identification of drug-target, gene-disease and drug-disease relationships using the EU-ADR corpus. A selection of the results obtained by BeFree by 10-fold cross-validation on the EU-ADR corpus and the performance of SemRep on the same corpus are shown. The first column indicates the number of the experiment as it appears in http://ibi.imim.es/befree/#supplbefree, Table 1. The second column shows if K SL is used with (TG+SBG) or without (TG) sparse bigrams, or if it is not used (-). The next two columns focus on K DEP walk features indicating the use of one of the following features: token (T), stem (S), lemma (L), POS-tag (P), role (R) or none (-). Finally, the last columns show the result obtained in each experiment indicating Precision (P), Recall (R) and f-measure (F) in percentage (%). *In the case of SemRep, note that the results were not obtained by cross-validation. [file 12859_2015_472_MOESM3_ESM.docx]

**Additional file 3: Table S2.**

| **BeFree** | | | | **EU-ADR** | | | | | | | | |
| --- | --- | --- | --- | --- | --- | --- | --- | --- | --- | --- | --- | --- |
| Experiment | ***K_SL_*** | ***K_DEP_*** | |  |  |  |  |  |  |  |  |  |
|  |  | *v-walk* | *e-walk* | Drug-Disease | | | Gene-Disease | | | Target-Drug | | |
|  |  |  |  | P | R | F | P | R | F1 | P | R | F |
| 1 | TG |  |  | 73.4 | 81.0 | 76.7 | 74.2 | 89.6 | 80.9 | 72.7 | 93.6 | 80.8 |
| 2 | TG+SBG |  |  | 72.9 | 79.7 | 75.7 | 73.6 | 89.7 | 80.5 | 72.7 | 94.1 | 81.1 |
| 3 | - | S | - | **70.2** | **93.2** | **79.3** | **75.1** | **97.7** | **84.6** | 72.9 | 97.4 | 82.5 |
| 5 | - | L | - | 70.2 | 92.5 | 79.0 | **75.1** | **97.7** | **84.6** | 72.9 | 97.4 | 82.5 |
| 19 | - | P | P | 74.5 | 71.5 | 72.3 | 74.7 | 53.0 | 59.5 | 72.2 | 67.6 | 68.9 |
| 21 | - | R | P | 72.6 | 70.2 | 70.5 | 77.9 | 53.0 | 61.7 | 75.2 | 68.1 | 70.2 |
| 30 | - | L | R | 66.8 | 65.1 | 63.6 | 83.8 | 71.0 | 75.6 | 71.3 | 73.1 | 70.8 |
| 75 | TG+SBG | L | - | 72.0 | 84.0 | 77.0 | 74.0 | 96.8 | 83.6 | 72.9 | 97.5 | 82.5 |
| 80 | TG+SBG | - | L | 71.9 | 82.1 | 76.3 | 73.8 | 94.8 | 82.7 | 73.0 | 98.0 | 82.8 |
| 102 | TG+SBG | T | R | 72.7 | 81.3 | 76.2 | 75.1 | 91.8 | 82.4 | **74.2** | **97.4** | **83.3** |
| **SemRep*** | | | | 100 | 40.0 | 57.0 | 96.0 | 36.0 | 52.0 | 95.0 | 39.0 | 55.0 |
